# Abdominal Wound Problems After Hysterectomy With Electrocautery vs. Scalpel Subcutaneous Incision

**DOI:** 10.1155/S1064744993000079

**Published:** 1993

**Authors:** David L. Hemsell, Patricia G. Hemsell, Brenda Nobles, Edward R. Johnson, Bertis B. Little, Molly Heard

**Affiliations:** 1 Departments of Obstetrics and Gynecology and Anesthesia The University of Texas Southwestern Medical Center Parkland Memorial Hospital Dallas TX USA; 2 UT Southwestern Medical Center at Dallas 5323 Harry Hines Blvd., G6.226 Dallas TX 75235-9032 USA

## Abstract

The purpose of this study was to evaluate the relationship between postoperative abdominal incision problems and opening subcutaneous tissues with electrocautery or scalpel. Women scheduled for elective abdominal hysterectomy who gave informed consent were randomly assigned to subcutaneous abdominal wall tissue incision by electrocautery or scalpel. Postoperative abdominal wound problem diagnoses included seroma, hematoma, infection, or dehiscence without identifiable etiology. Fifteen of 380 women (3.9%) developed a wound problem; six had scalpel and nine had electrosurgical subcutaneous incisions (*P* = 0.4). Thicker subcutaneous tissues (*P* = 0.04) and concurrent pelvic infection (P < 0.001) were significant risk factors for postoperative wound problems. Only two women (0.5%) developed an infection. We conclude that the method of subcutaneous tissue incision was unrelated to the development of postoperative abdominal incision problems in 380 women undergoing elective abdominal hysterectomy.


Infectious Diseases in Obstetrics and Gynecology 1:27–31 (1993)
© 1993 Wiley-Liss, Inc.

### Abdominal Wound Problems After Hysterectomy With Electrocautery vs. Scalpel Subcutaneous Incision

**David L. Hemsell, Patricia G. Hemsell, Brenda Nobles, Edward R. Johnson, Bertis B. Little, and Molly Heard**

*Departments of Obstetrics and Gynecology and Anesthesia, The University of Texas Southwestern Medical Center, Parkland Memorial Hospital, Dallas, TX*

ABSTRACT

**The purpose of this study was to evaluate the relationship between postoperative abdominal incision problems and opening subcutaneous tissues with electrocautery or scalpel. Women scheduled for elective abdominal hysterectomy who gave informed consent were randomly assigned to subcutaneous abdominal wall tissue incision by electrocautery or scalpel. Postoperative abdominal wound problem diagnoses included seroma, hematoma, infection, or dehiscence without identifiable etiology. Fifteen of 380 women (3.9%) developed a wound problem; six had scalpel and nine had electrosurgical subcutaneous incisions ($P = 0.4$). Thicker subcutaneous tissues ($P = 0.04$) and concurrent pelvic infection ($P < 0.001$) were significant risk factors for postoperative wound problems. Only two women (0.5%) developed an infection. We conclude that the method of subcutaneous tissue incision was unrelated to the development of postoperative abdominal incision problems in 380 women undergoing elective abdominal hysterectomy.** © 1993 Wiley-Liss, Inc.

KEY WORDS

Postoperative, nonunion/infection, trees, plane, desert

An electrosurgical knife was reported to be one of several variables contributing to postoperative abdominal wall incision infection.[1] A randomized clinical trial was designed to evaluate the relationship between postoperative abdominal incision infection or separation due to any cause, and dividing subcutaneous abdominal wall tissues with either a scalpel or electrocautery prior to elective abdominal hysterectomy. The incidence and type of wound problems and risk factors would be determined.

### MATERIALS AND METHODS

Women scheduled to undergo elective abdominal hysterectomy for benign disease were invited to participate in this clinical trial and to sign an Institutional Review Board-approved consent form. They were assigned to the scalpel or electrocautery group by a computer-generated randomization code. Exclusion criteria included pregnancy, pelvic malignancy, allergy to cephalosporin or history of immediate hypersensitivity reaction to penicillin, requirement for other antimicrobials, and history of receiving antibiotics during the preceding 7 days. A medical history was taken, a complete physical examination was performed, and routine laboratory tests were done. A povidone iodine shower and douche were taken by the patients the night prior to surgery. They were given a single 1 g preoperative dose of cefazolin.

The abdominal skin and vagina were prepped with povidone iodine in the operating room after general anesthesia was administered, and the surgi-

**Address correspondence/reprint requests to Dr. David L. Hemsell, UT Southwestern Medical Center at Dallas, 5323 Harry Hines Blvd., G6.226, Dallas, TX 75235-9032.**

Received November 17, 1992

Accepted February 11, 1993

cal procedures were performed by third year house officers under faculty supervision. The abdominal skin was incised with a scalpel. Separate scalpel blades were used for skin and subcutaneous tissues in women in that arm of the study, cut vs. burn. Subcutaneous tissues were opened by either scalpel or electrocautery using the pure cut mode with an SSE2 L (Valleylab, Boulder, CO) electrosurgical generator. Physicians selected the power settings that were used. For the SSE2 L machine, settings were numbered, i.e., 3, 4, 5. Each setting delivered a fixed amount of monopolar current at a 500 ohm load. For example, the setting for 3 was to deliver 75 watts, for 4 it was 125 watts, for 5 it was 160 watts, etc. Subcutaneous tissue thickness was measured at the maximal depth prior to opening the fascia. Hemostasis of individual bleeding sites could be accomplished with electrocautery or suture in either group. The pelvis was irrigated with sterile saline following the hysterectomy, as were the subcutaneous tissues after fascial closure. For uniformity, subcutaneous tissues were not closed. Skin edges were approximated with staples. The study subjects were evaluated clinically twice daily during the immediate postoperative period, and were followed for up to 6 weeks after discharge from the hospital to detect the occurrence of both early and late wound problems. A wound culture was performed if incisional separation occurred irrespective of etiology.

Several categories of wound problems were included. *Dehiscence* was defined as separation of the subcutaneous tissues without seroma, hematoma, erythema, induration, unexpected tenderness, purulent secretions, or a positive bacteriologic culture. *Seroma* was the diagnosis when there was incisional dehiscence with abundant serous fluid. *Hematoma* was defined when it was discovered in the wound at incisional dehiscence. Volume of serous fluid was not measured nor were hematomas. *Wound infection* was diagnosed if erythema, induration, and unexpected tenderness were present, if there was purulence at the time of dehiscence, or if there was a positive bacteriologic culture from that space. Quetelet's index [weight (kg)/height$^2$(m)], was computed.

Data were analyzed using Student's $t$ test, Fisher's exact probability test, Mann-Whitney $U$ (Wilcoxon) statistic, Welch's approximation, or log likelihood Chi-square tests and by multiway analysis of variance.

### RESULTS

Between July, 1987 and November, 1988, 380 women were enrolled in this clinical trial. The indications for surgery, similar in the two patient populations, were uterine leiomyoma (54%), chronic pelvic pain (16%) or abnormal bleeding (10%) unresponsive to medical management, stress urinary incontinence (8%), adnexal mass (7%), and cervical intraepithelial neoplasia plus abnormal uterine bleeding (5%). There was no significant correlation between the preoperative diagnosis and the development of a postoperative wound problem. Sixty-two percent of the incisions were vertical, and the remainder were low transverse. The direction of abdominal wall incision was evenly distributed in women with each type of subcutaneous incision.

The clinical and surgical variables were similar in the two groups of women with the exception of race (Table 1). The distribution of wound problems is presented in Table 2. Demographic, surgical, and laboratory variables for these women are presented in Table 3 and are compared with those of women without wound problems. Race was not significantly related to the development of a wound problem following surgery. There were no cases of fascial dehiscence. More women whose subcutaneous tissues were opened with cautery did develop wound problems, but this difference was not statistically significant ($P = 0.4$).

Diabetes and asthma were not risk factors for wound problems, and no women were receiving steroids. Pelvic infection was a risk factor. Twenty-eight women (7.4%) developed a postoperative pelvic infection requiring parenteral antimicrobial therapy; 13 of these had scalpel and 15 had electrocautery subcutaneous entry ($P = 0.7$). The mean hospital stay for these 28 women was 8.7 days, significantly ($P < 0.001$) prolonged over the entire group, as would be expected. Hospital stay associated with any type of wound problem was similar ($P = 0.8$), and was 2 days longer than that observed for women with pelvic infection only ($P < 0.001$). Heavier women had deeper subcutaneous tissues, which increased significantly ($P = 0.04$) the likelihood of developing a postop-

**TABLE 1. Clinical and surgical variables**

| Clinical/surgical variables | Subcutaneous incision (mean ± SD) Scalpel (n = 191) | Electrocautery (n = 189) | Significance (P) |
|---|---|---|---|
| Age (years) | 39 ± 8.8 | 39 ± 8.3 | 1.0 |
| Height (inches) | 63 ± 3.2 | 63 ± 3.3 | 1.0 |
| Weight (pounds) | 169 ± 45.4 | 168 ± 41.3 | 0.8 |
| Race | | | 0.04 |
| Black | 122 | 101 | |
| White | 44 | 66 | |
| Hispanic | 20 | 21 | |
| Other | 5 | 1 | |
| Preoperative hemoglobin (g/dL) | 12.1 ± 1.8 | 12.2 ± 1.5 | 0.1 |
| Postoperative hemoglobin (g/dL) | 10.4 ± 1.7 | 10.5 ± 1.7 | 0.4 |
| Operation duration (min) | 137 ± 42.4 | 137 ± 39.9 | 1.0 |
| Incision depth (cm) | 3.5 ± 1.7 | 3.6 ± 1.7 | 0.9 |
| Hospital stay (days) | 4.7 ± 1.9 | 4.6 ± 1.5 | 0.6 |

**TABLE 2. Wound problems**

| Problem | Subcutaneous incision Scalpel | Electrocautery |
|---|---|---|
| Infection | — | 2 |
| Seroma | — | 1 |
| Hematoma | 3 | 2 |
| Dehiscence | 3 | 4 |

erative wound problem. This applied to women with vertical and horizontal incisions. When evaluated by multiway analysis of variance, with subcutaneous depth as the dependent variable, increased age ($P = 0.01$) and Quetelet's index ($P = 0.0001$) were important, but the type of incision (horizontal or vertical) was not related ($P = 0.2$) to the development of a postoperative wound problem.

### DISCUSSION

Although abdominal wall infection after hysterectomy has been addressed, the incidence of abdominal incisional dehiscence without infection is generally unreported. Dehiscence without infection was observed 6.5 times more frequently than was that associated with infection in this study, and the mean hospital stay was similar. The dose of prophylactic antibiotic administered to the women in this trial might theoretically lower the postoperative infection rate in the abdominal wall, and decrease the potential for recovering bacteria from a clinically uninfected but colonized wound.

The association between wound problems and pelvic infection is definite, but without established cause. It is possible that the number of bacterial colonies was low and was undetected by culture, but there were no clinical signs of infection. There was no obvious impairment of host-defense mechanisms in any of these women. Such impairment is more likely to occur among older and heavier women, however, and a higher incidence of pelvic infection may also be consistent with lowered host immunocompetence. None of the older, heavier women in this clinical trial had diabetes. Only one of these women had a seroma, the etiology of which is also unknown. Although there was no evidence of subcutaneous bleeding prior to skin closure in any of the women, bleeding apparently occurred postoperatively in at least five women, and may be ascribed as the etiology for their postoperative wound separation. The largest group of women (seven) with a wound problem had neither an excessive amount of serous fluid, hematoma formation, clinical evidence of infection, nor positive bacteriologic culture. The role of the dose of prophylactic antibiotic in this scenario is unknown. Mean prolongation of the hospital stay by the development of a wound problem was 5.2 days, more than a twofold increase, emphasizing the importance of wound problems and their impact on health care costs. Unexpectedly, separation of the incision without infection had the same impact on hospital stay as did wound infection. Utilization of

**TABLE 3. Variables for women without and with postoperative abdominal incisional problems[a]**

| Variable | No wound problem (n = 365) | Wound problem (n = 15) | Significance (*P*) |
|---|---|---|---|
| Age (years) | 39.6 ± 8.6 | 39.6 ± 8.1 | 1.0 |
| Height (inches) | 63.6 ± 3.0 | 65.5 ± 3.6 | 0.02 |
| Weight (pounds) | 168.2 ± 42.0 | 187.6 ± 68.4 | 0.5 |
| Quetelet's index (kg/m$^2$) | 29.4 ± 7.1 | 30.9 ± 10.8 | 0.4 |
| Race | | | 0.8 |
| Black | 214 | 9 | |
| White | 105 | 5 | |
| Hispanic | 41 | 1 | |
| Other | 5 | 1 | |
| Diabetes | 27 | 0 | 0.3 |
| Asthma | 14 | 1 | 0.5 |
| Preoperative hemoglobin (g/dL) | 12.2 ± 1.7 | 12.2 ± 0.9 | 0.9 |
| Postoperative hemoglobin (g/dL) | 10.5 ± 1.7 | 10.4 ± 1.7 | 0.9 |
| Electrocautery power setting (watts @ 500 ohm load) | 128.5 ± 28.2 | 117.8 ± 30.9 | 0.4 |
| Subcutaneous depth (cm) | 3.5 ± 1.7 | 4.1 ± 2.1 | 0.04 |
| Operation duration (minutes) | 136.7 ± 41.0 | 154.0 ± 41.3 | 0.1 |
| Hospital stay (days) | 4.5 ± 1.1 | 9.7 ± 3.9 | <0.001 |
| Pelvic infection | 22 | 6 | <0.001 |

[a]Data are mean ± standard deviation.

visiting nurses might shorten the hospital stay of women with wound problems.

The SSE2 L unit has been replaced in our operating rooms by a Force 2 (Valleylab) electrosurgical generator. Whereas the mean power setting of the SSE2 L observed in this clinical trial was over 117 watts, only 30 to 40 watts are required to achieve the same result with the new model. This is made possible by new software and hardware, alternations in peak to peak voltage, altered rated load, more power at lower tissue impedance, and modified power curves.

Prior to this clinical investigation, the general practice in Parkland Memorial Hospital was to close the subcutaneous space with interrupted or running plain catgut suture after abdominal hysterectomy. A scalpel was used for the subcutaneous incision. Abdominal incision infection rates were as high as 6% in retrospective evaluations without prophylaxis and in prospective evaluations. For uniformity in this trial, subcutaneous suture was not placed, and the infection rate with prophylaxis was only 0.5%. That difference in infection rate, by historical comparison, is highly significant ($P < 0.001$), indicating that foreign bodies contribute to wound infection, as indicated by Ledger[1] and many others.

Cruse[2] reported that use of the electrosurgical knife almost doubled the infection rate in all four categories of wound (clean, clean contaminated, contaminated, and dirty). When electrosurgical technology was new and techniques were unrefined, large amounts of charred tissue were frequently left in the wound. This was believed to act as a foreign body, which enhanced wound infection when combined with necrotic tissue resulting from thermal injury. Cruse and Foord[3] later reported that with operating room experience, and reduced generalized tissue destruction and char, the infection rate in patients with clean wounds was the same whether or not the electrosurgical generator was used. Prophylactic antibiotic was not administered to those patients as it was in the women presented here.

Johnson and Serpell[4] reported no difference in wound inflammation or infection rates in 240 general surgical patients whose incisions were made with scalpel or electrocautery. Their patients also received prophylaxis, and patients with all categories of wounds were included, in contrast to the present report. Thirty-five percent of patients included in that report had contaminated wounds, from which arose 71% of inflamed incisions and 81% of the wound infections, however.

There are animal model data documenting slower wound healing after electrosurgical incision when compared with scalpel incision. In such a report by Arnaud and Adloff,[5] there were comparable bursting strengths. In addition to the incision, electrocautery results in adjacent tissue necrosis.[6] Madden and coinvestigators[7] reported an increased incidence of, and susceptibility to, infection after electrocautery incision when compared with scalpel incision in an albino guinea pig model. It was correlated with histologic evidence of delayed healing and tissue coagulation necrosis.

The power of the data generated during this clinical trial did not detect a statistically significant difference in infection or other wound problems between women whose subcutaneous tissues were opened with an electrosurgical generator or a scalpel. There were 50% more adverse wound outcomes in women whose tissues were opened with an electrosurgical generator, however. It would require a sample size that was six times larger than that reported here, and a constant prevalence of wound problems in both groups, to detect a significant ($P = 0.046$) difference in wound complications. The estimated number of abdominal hysterectomies performed in women in the United States for 1990 was 430,000.[8] If one-half of those procedures included opening subcutaneous tissues with an electrosurgical instrument, and the same percentage of women had wound problems, an anticipated 18,000 additional hospital days would result from wound problems associated with electrosurgical entry.

In addition, Gatti and coworkers[9] detected mutagenicity of electrocautery smoke collected during mammoplasty; the testing was performed by the Hazard Evaluations and Technical Assistance Branch of the National Institute of Occupational Safety and Health (NIOSH). The impact on operating room personnel is unknown, but it does appear that this surgical technique has the capacity for adverse reaction. Risks to the patient and operating room personnel may outweigh the hemostatic benefit of electrocautery incision.

### REFERENCES

1. Ledger W: Prevention, diagnosis, and treatment of postoperative infections. Obstet Gynecol 55(Suppl 5):203S–206S, 1980.
2. Cruse PJE: Some factors determining wound infection. A prospective study of 30,000 wounds. In Polk HC Jr, Stone HH (eds): Hospital-Acquired Infections in Surgery. Baltimore: University Park Press, pp 79–85, 1977.
3. Cruse PJE, Foord R: The epidemiology of wound infection. A 10-year prospective study of 62,939 wounds. Surg Clin North Am 60:27–40, 1980.
4. Johnson CD, Serpell JW: Wound infection after abdominal incision with scalpel or diathermy. Br J Surg 77:626–627, 1990.
5. Arnaud JP, Adloff M: Electrosurgery and wound healing: An experimental study in rats. Eur Surg Res 12:439–443, 1980.
6. Mitchell JP, Lumb GN, Dobbie AK: A Handbook of Surgical Diathermy. Bristol: John Wright, pp 8–9, 1978.
7. Madden JE, Edlich RF, Custer JR, Panek PH, Thul J, Wangensteen OH: Studies in the management of the contaminated wound. IV. Resistance to infection of surgical wounds made by knife, electrosurgery and laser. Am J Surg 119:219–222, 1970.
8. Detailed Diagnosis and Procedures National Hospital District Survey, National Center for Health Statistics: Health, United States, 1990. DHHS Pub. No. (PHS) 91-1232. Hyattsville, MD: Public Health Service, 1991.
9. Gatti JE, Bryant CJ, Noone RB, Murphy JB: The mutagenicity of electrocautery smoke. Plast Reconstr Surg 89: 781–784, 1992.
